# A Simple Methodology for Conversion of Mouse Monoclonal Antibody to Human-Mouse Chimeric Form

**DOI:** 10.1155/2013/716961

**Published:** 2013-09-02

**Authors:** Vinh T. Dang, Kedar D. Mandakhalikar, Oi-Wing Ng, Yee-Joo Tan

**Affiliations:** ^1^Infrastructure, Technology & Translational Division, Institute of Molecular and Cell Biology (IMCB), Agency for Science,Technology and Research, 61 Biopolis Drive, Singapore 138673; ^2^Department of Microbiology, Yong Loo Lin School of Medicine, National University Health System (NUHS), National University of Singapore, Singapore 117597

## Abstract

Passive immunotherapy has mainly been used as a therapy against cancer and inflammatory conditions. Recent studies have shown that monoclonal antibody-(mAb-) based passive immunotherapy is a promising approach to combat virus infection. Specific mouse mAbs can be routinely generated in large amounts with the use of hybridoma technology but these cannot be used for therapy in human beings due to their immunogenicity. Therefore, the development of chimeric and humanized mAbs is important for therapeutic purpose. This is facilitated by a variety of molecular techniques like recombinant DNA technology and the better understanding of the structure and function of antibody. The human-mouse chimeric forms allow detailed analysis of the mechanism of inhibition and the potential for therapeutic applications. Here, a step-by-step description of the conversion process will be described. The commercial availability of the reagents required in each step means that this experimentation can be easily set up in research laboratories.

## 1. Introduction

Emil von Behring (1854–1917) won the first Nobel Prize in medicine for demonstrating that humoral immunity could be transferred from immunized animals to humans. Using heterologous sera in humans had its own limitations because of immunological reactions to serum proteins, for example, hypersensitivity. With the help of techniques for better purification of antibodies and monoclonal antibody (mAb) engineering, we now have overcome many of these complications and have attained improved specificity. Till recently, the main focus of the use of the recombinant mAb and passive immunotherapy had been for treatment of cancers or inflammatory conditions [1, 2].

MAb-based immunotherapy is becoming important in infectious diseases because of widespread resistance to drugs among pathogens, immunocompromised hosts, and the emergence of new pathogens. For controlling pathogens such as acute cytopathic viruses that can cause fatal damage in infected tissues, the best way is to prevent the disease. As vaccination is not always available or suitable, passive immunotherapy could be used to provide protection in the periods of high-exposure risk [3]. As immunotherapy is a promising approach to combat virus infection, much research efforts have been devoted to the generation and characterisation of virus-neutralizing mAbs [4–6]. In many laboratories, hybridoma clones are derived from mouse or rat B-lymphocytes by fusion with myeloma cell line (e.g., SP2/0, NS0, NS1, Ag8, or P3U1) [7]. One major limitation of using these hybridoma-derived mAbs is that human-anti-mouse or human-anti-rat antibody response can occur as a result of immunogenicity of these mouse or rat antibodies [4]. Therefore, it is important to humanize these antibodies for human therapeutic purposes without impacting their binding affinity towards antigen targets. For example, in virus research, after a mouse mAb is selected for its potent virus neutralizing activity, it will be useful to convert it into human-mouse chimeric form. If the human-mouse chimeric form has similar neutralizing activity; this will be a reason for further development for therapeutic application. Hence, the technique for converting mouse mAb into human-mouse chimeric form is an emerging research tool.

Chimeric antibody has been successfully produced and tested for specific binding activity in many previous studies [8–12]. For example, chimeric anti-human DR5 MAb (cmDRA6) can bind to DR5 antigen as demonstrated by both ELISA and Western blot [10]. In addition, a human-mouse chimeric antibody generated from mAb against hepatitis E virus (HEV) capsid proteins E2 still maintains binding activity similar to the original mAb as shown by ELISA and Western blot [11]. Since chimeric antibody is expected to be less immunogenic in human, it could be suitable for antibody therapy of viral infections. Indeed, it has been demonstrated that patient, who received chimeric antibody 17-1A, did not show any toxic or allergic reactions and the chimeric antibody appears significantly less immunogenic than its parental murine antibody [13]. The construction of human-mouse chimeric antibody basically involves cloning and ligating of the variable region genes of mouse mAbs into expression vectors, which have heavy- and light-chain immunoglobulin constant regions. A simple methodology for this conversion will be described here in a step-by-step manner.

## 2. Results and Discussion

The first step is to amplify heavy- and light-chain immunoglobulin variable regions (*V*_*H*_ and *V*_*L*_) using the polymerase chain reaction (PCR). To obtain first-strand cDNA for PCR reaction, messenger RNA (mRNA) needs to be extracted from hybridoma cells before reverse transcription. The success to obtain a correct sequence for each *V*_*H*_ and *V*_*L*_ mainly depends on the selection of primer set and optimised conditions of PCR reaction. Different primer sets have been developed for amplifying the variable domains [14–17]. Mouse Ig-Primer set is also commercially available. For example, the one from Novagen has been successfully applied in many previous studies (e.g., [18–21]).

In order to determine the sequence of DNA products from PCR, blunt end ligation with a DNA topoisomerase provides an efficient way to clone the DNA into a vector [22]. While the use of polymerase with proofreading activity significantly reduces the chance of mutations being introduced in the PCR amplification step, the sequence alignment of multiple clones will also easily reveal a mismatch that is found in a rare clone. In most of the clones, an identical sequence should be obtained. However, if two or more sets of sequences are obtained, then, it is very likely that the hybridoma cells used for mRNA extraction are heterogeneous, implicating that hybridoma cells need to be further subcloned.

**Figure 1:**
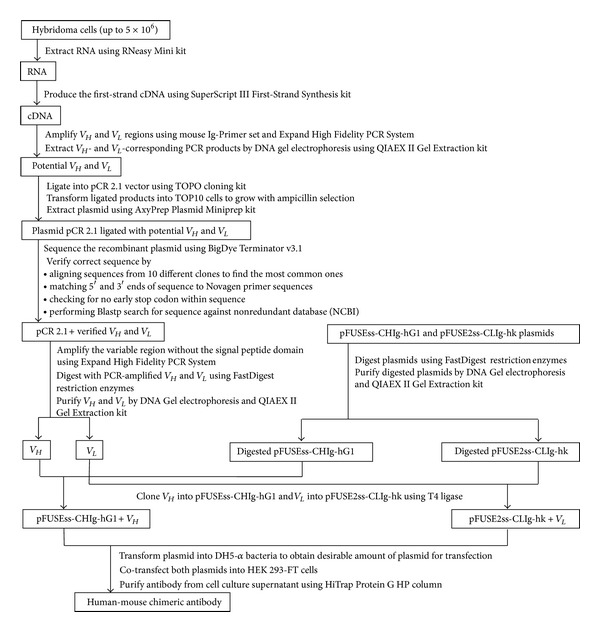
Basic molecular procedure for converting mouse mAbs to human-mouse chimeric forms.

Once the correct *V*_*H*_ and *V*_*L*_ sequences have been obtained, they can be subcloned into vectors containing the constant region of the human heavy and light chain, respectively (Figure 1). These vectors are designed for mammalian expression and can be home-made or commercially available. An example of the latter is the pair of pFUSEss-CHIg-hG1 and pFUSE2ss-CLIg-hk vectors from InvivoGen. Expression of the human-mouse chimeric antibody is achieved by transfecting the vectors into a transfectable mammalian cell line like the Human Embryonic Kidney 293 cells (HEK 293). The secreted antibody can then be purified from the culture supernatant and tested to determine if it still retains the antigen-binding capacity after chimerization. A direct comparison with the mouse monoclonal antibody produced by the hybridoma will reveal if there is any incompatibility between the mouse variable and human constant regions in the mouse-human chimeric antibody.

**Figure 2:**
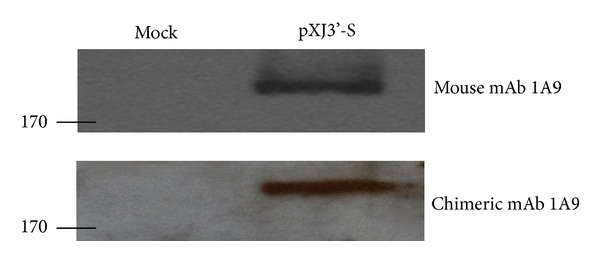
Western blot analysis for the detection of SARS-CoV spike (S) protein (210 kDa). HEK 293-FT cells were transfected with no plasmid (mock) or with plasmid expressing full-length S, pXJ3′-S. Cell lysates were separated in a 7.5% SDS-PAGE gel and detected using mouse mAb 1A9 and human-mouse chimeric mAb 1A9 via Western blot analysis as previously described [23]. Molecular weight markers (in kilodaltons) are indicated on the left.

The previous methodology is summarized in Figure 1 and has been used to convert the mouse mAb 1A9 to its chimeric human-mouse form. mAb 1A9 is an antibody generated in mice against the severe acute respiratory syndrome coronavirus (SARS-CoV) spike protein [23]. As shown in Figure 2, both the mouse 1A9 and chimeric 1A9 are able to bind to spike protein in HEK 293-FT cell lysates that were transfected with plasmid containing the full-length spike gene, pXJ3′-S.

## 3. Materials and Methods

### 3.1. Amplification of *V*_*H*_ and *V*_*L*_ Regions

- Count and collect up to 5 × 10^6^ hybridoma cells by centrifugation (800 g for 5 minutes). Wash cells twice with PBS and remove the supernatant completely. Store pelleted cells at −20°C if not used immediately.
- Extract RNA from hybridoma cells using RNeasy Mini Kit (QIAGEN). Suspend the cells in 350 *μ*L lysis buffer by vortexing and pass through QIAshredder (QIAGEN) spin column for RNA extraction.
- Produce the first-strand cDNA from the RNA using SuperScript III First-Strand Synthesis kit (Invitrogen). Use 1 *μ*L Novagen mouse 3′ primer (Mouse Ig-Primer set, Novagen) in total of 20 *μ*L reaction volume (Note 1).
- Amplify *V*_*H*_ and *V*_*L*_ regions using Expand High Fidelity PCR System (Roche) (Note 2). Add Novagen 5′ A-B leader primers to final concentration of 10 pmol *μ*L^−1^ and C-G leader primers to 5 pmol *μ*L^−1^. Optionally, add Novagen 3′ primer to reach final concentration of 5 pmol *μ*L^−1^.
- Run DNA gel electrophoresis on a 2% agarose gel for PCR products. Extract the DNA bands at around 500 bp (Note 3), as determined by GeneRuler 100 bp DNA Ladder (Thermo Scientific), using QIAEX II Gel Extraction Kit (QIAGEN). Elute DNA in nuclease-free water.

### 3.2. Cloning Potential *V*_*H*_ and *V*_*L*_ into Vector for Sequencing

- Ligate potential *V*_*H*_- and *V*_*L*_-amplified products after gel extraction into pCR 2.1 vector from TOPO cloning kit (Invitrogen). Transform ligated products into competent TOP10 cells by heat shock method. Add transformed bacteria onto LB agar plates, which contain 100 *μ*g mL^−1^ ampicillin (Sigma) and 40 *μ*L X-gal solution (Thermo Scientific). Incubate plates overnight at 37°C.
- Grow bacteria from at least 10 white or light blue colonies in LB broth containing 100 *μ*g mL^−1^ ampicillin for up to 16 hours at 37°C.
- Collect up to 4 mL bacteria for plasmid extraction using AxyPrep Plasmid Miniprep Kit (Axygen Biosciences). Keep bacteria in LB broth at 4°C for short-term storage (up to 4 weeks).
- Cut recombinant plasmid (5–10 *μ*L) by FastDigest restriction enzyme EcoRI (Thermo Scientific) and run the DNA gel electrophoresis to confirm the presence of DNA insert.
- Sequence the recombinant plasmid using BigDye Terminator v3.1 (Applied Biosystems) with M13 forward primer (5′-GTAAAACGACGGCCAG-3′) or M13 reverse primer (5′-CAGGAAACAGCTATGAC-3′).
- Verify correct sequence by (1) aligning sequences from 10 different clones to find the most common ones; (2) matching 5′ and 3′ ends of sequence to Novagen primer sequences; (3) checking for no early stop codon within sequence; (4) performing Blastp search for sequence against nonredundant database (NCBI) (Note 4).

### 3.3. Cloning *V*_*H*_ and *V*_*L*_ into Expression Vector

- Transform pFUSEss-CHIg-hG1 and pFUSE2ss-CLIg-hk plasmids (InvivoGen) separately into *E. coli* DH5-*α* competent cells (Invitrogen) to obtain desirable amount of plasmid for subsequent digestion and ligation steps. Pick up one transformed colony from overnight-incubated plate and grow it in LB broth with appropriate antibiotic (25 *μ*g mL^−1^ zeocin (InvivoGen) or 50 *μ*g mL^−1^ blasticidin S HCl (InvivoGen) up to 16 hours at 37°C. Collect up to 100 mL bacteria culture for plasmid extraction using Midiprep kit (QIAGEN).
- Identify the signal peptide at 5′ end of the *V*_*H*_ and *V*_*L*_ by SignalP (http://www.cbs.dtu.dk/services/SignalP/). Amplify the variable region without the signal peptide domain using Expand High Fidelity PCR System. Forward and reverse primers have 15–20 bp overlap with the appropriate regions within *V*_*H*_ or *V*_*L*_ plus 6 bp at 5′ end as restriction sites (e.g., EcoRI and NheI for *V*_*H*_, EcoRI and BsiWI for *V*_*L*_, Note 5) and 2–4 bp before restriction sites to ensure efficient digestion by FastDigest restriction enzymes (Thermo Scientific). It is important to ensure no frameshifting after the *V*_*H*_ or *V*_*L*_ is ligated into pFUSE vectors.
- Digest pFUSEss-CHIg-hG1 and PCR-amplified *V*_*H*_ separately using the same FastDigest restriction enzymes (e.g., EcoRI and NheI) and pFUSE2ss-CLIg-hk plasmids and PCR-amplified *V*_*L*_ separately using the same FastDigest restriction enzymes (e.g., EcoRI and BsiWI).
- Run DNA Gel electrophoresis on 1% agarose gel to purify the digested plasmids or PCR products and extract the DNA by using QIAEX II Gel Extraction Kit. Elute DNA in nuclease-free water.
- Clone *V*_*H*_ into pFUSEss-CHIg-hG1 and *V*_*L*_ into pFUSE2ss-CLIg-hk using T4 ligase enzyme (Thermo Scientific). The molar ratio of DNA insert to vector for ligation reaction is 3 : 1 (Note 6).
- Transform the plasmid after ligation step into DH5-*α* competent cells by heat shock method. Grow pFUSEss-CHIg-hG1 with *V*_*H*_ on LB agar plates containing 25 *μ*g mL^−1^ zeocin and pFUSE2ss-CLIg-hk with *V*_*L*_ on LB agar plates containing 50 *μ*g mL^−1^ blasticidin S HCl overnight at 37°C. Pick up several transformed colonies from plate and grow them in LB broth with appropriate antibiotic (25 *μ*g mL^−1^ zeocin or 50 *μ*g mL^−1^ blasticidin S HCl).
- Collect up to 4 mL bacteria for plasmid extraction using AxyPrep Plasmid Miniprep Kit. Keep bacteria in LB broth at 4°C for short-term storage (2–4 weeks).
- Cut recombinant plasmid (5–10 *μ*L) by FastDigest restriction enzymes and run the DNA gel electrophoresis to confirm the presence of DNA insert.
- Optional step: sequence the DNA insert in the recombinant plasmid using BigDye Terminator v3.1.
- Grow bacteria with correct recombinant plasmid in LB broth with appropriate antibiotic (25 *μ*g mL^−1^ zeocin or 50 *μ*g mL^−1^ blasticidin S HCl) up to 16 hours at 37°C. Collect up to 100 mL bacteria culture for plasmid extraction using Midiprep kit.

### 3.4. Antibody Expression and Purification

- Seed 1.5 million of Human Embryonic Kidney 293-FT cells (HEK 293-FT) (Invitrogen) in 6 cm dish (Nunc) with 3 mL transfection media (Dulbecco's Modified Eagle's Medium (DMEM) (Invitrogen) and 10% fetal bovine serum (Hyclone)) for overnight at 37°C and 5% CO_2_ (Note 7).
- Add 2 *μ*g of each recombinant plasmid (pFUSEss-CHIg-hG1 with *V*_*H*_ and pFUSE2ss-CLIg-hk with *V*_*L*_) and 12 *μ*L Lipofectamine 2000 (Invitrogen) to separate tubes containing 100 *μ*L Opti-Mem (Invitrogen). Vortex briefly and incubate at room temperature (RT) for 5 minutes.
- Spin briefly and mix Opti-Mem with plasmid and Lipofectamine together into the same tube. Incubate for 20 minutes at RT.
- Aspirate cell culture media and gently add 1.5 mL of transfection media with mixture of Lipofectamine and plasmid (Note 8).
- Swirl dish gently to mix and incubate for 4–6 hours at 37°C and 5% CO_2_.
- Gently aspirate media from the dish. Add 3 mL fresh transfection media and incubate at 37°C and 5% CO_2_.
- Collect the cell culture supernatant three times after every 24 hours with replenishment of fresh transfection media each time. Keep cell culture supernatant at 4°C if not used immediately.
- Purify antibody from the cell culture supernatant using HiTrap Protein G HP 1 mL column (GE Healthcare) (Note 9).
- Run the Bradford protein assay (Bio-Rad) with standard BSA to determine protein concentration in each fraction.
- Run 3–5 eluted fractions with the highest protein concentration on 12% SDS-PAGE and stain gel with coomassie blue dye to visualise Ig heavy and light chains (approximately 56 kDa and 26 kDa, resp.).

## 4. Notes

1. Use MuIgM*V*_*H*_3′-1 for heavy chain IgM, MuIgG*V*_*H*_3′-2 for heavy chain IgG, MuIg*κV*_*L*_3′-1 for light chain kappa, and MuIg*λV*_*L*_3′-1 for light chain lambda during first-strand cDNA synthesis. If using Oligo (dT) primer to produce the first-strand cDNA, the Novagen 3′ primer must be added in PCR reaction.
2. Decrease the annealing temperate by steps of two degrees if no PCR products are found. Increase the annealing temperature by steps of two degrees if many non-specific PCR products are found.
3. Multiple bands may be present on DNA gel per reaction but purify DNA from band of expected size only (approximately 500 bp). Depending on hybridoma source, two PCR products for *V*_*L*_ region may be obtained since one is from myeloma cells [24].
4. Alternatively, IgBLAST can be used to verify correct sequence of *V*_*H*_ and *V*_*L*_ (http://www.ncbi.nlm.nih.gov/igblast/index.html).
5. Restriction sites introduced to forward and reverse primers for amplifying *V*_*H*_ are chosen based on restriction sites of pFUSEss-CHIg-hG1 in the 5′ to 3′ direction, including EcoRI, EcoRV, XhoI, and NheI. Restriction sites introduced to forward and reverse primers for amplifying *V*_*L*_ are chosen based on restriction sites of pFUSE2ss-CLIg-hk in the 5′ to 3′ direction, including EcoRI, AgeI, BstEII, NcoI, and BsiWI. Avoid choosing the restriction site if it is also found within *V*_*H*_ or *V*_*L*_ sequence because short fragment of *V*_*H*_ or *V*_*L*_ after digestion can be ligated into the vector.
6. Other molar ratios of insert DNA to vector, such as 5 : 1 and 1 : 1, can also be performed at the same time.
7. After overnight growth, HEK 293-FT cells should be around 80–90% confluent for optimal transfection.
8. Since the HEK 293-FT cells can be easily dislodged, the media should be gently aspirated and added to the same edge side of the dish.
9. The following protocol can be alternatively used to purify antibody from cell culture supernatant using HiTrap Protein G HP column:
  - attach HiTrap Protein G HP 1 mL column to a peristaltic pump and equilibrate by passing through 10–20 mL PBS at flow rate of less than 2 mL per minute,
  - filter the culture supernatant through 0.45 *μ*m filter to remove any cell debris and dilute the filtrate with one volume of PBS,
  - pass the diluted filtrate and then 10–20 mL PBS through the HiTrap column at flow rate of 1 mL per min,
  - elute antibody using elution buffer 0.05 M Glycine-HCl, pH 2.7,
  - collect and check pH of each 0.5 mL fraction,
  - add 2.5 *μ*L of 5 M NaOH for fraction with pH at around 2.7 to return pH to 7-8.
